# Multiple Approaches for Individualized Fertility Protective Therapy in Cancer Patients

**DOI:** 10.1155/2012/961232

**Published:** 2011-12-29

**Authors:** I. Demeestere, F. Moffa, F. Peccatori, C. Poirot, E. Shalom-Paz

**Affiliations:** ^1^Fertility Clinic, Universite Libre de Bruxelles (ULB), Hôpital Erasme, 1070 Brussels, Belgium; ^2^Fertility Clinic, Universite Libre de Bruxelles (ULB), Campus Erasme, 1070 Brussels, Belgium; ^3^Instituto Marquès, 08034 Barcelona, Spain; ^4^Department of Medicine, Division of Hematology Oncology, European Institute of Oncology, 20141 Milan, Italy; ^5^UF de Biologie de la Reproduction, Groupe Hospitalier Pitié-Salpêtrière, 75651 Paris, France; ^6^Department of Obstetrics and Gynecology, McGill University, Montreal, QC, Canada H3A 2T5

## Abstract

In the last decade, fertility preservation has risen as a major field of interest, creating new interactions between oncologists and gynecologists. Various options, such as cryopreservation of ovarian tissue, have been developed and are currently routinely proposed in many centers. However, many of the options remain experimental and should be offered to patients only after adequate counseling. This paper addresses the efficiency and the potential of the different fertility preservation approaches.

## 1. Introduction

Concurrent with the progress over the last few decades in oncology, the concept of fertility preservation has gained attention, driving new research endeavors. In the UK, approximately one out of every 10 cancers occurs in adults aged 15 to 49 years (Cancer Research UK statistics) and currently, one out of every 715 adults is a survivor of childhood cancer [1].

The new approaches to fertility preservation include cryopreservation and transplantation of ovarian tissue, follicular culture, pharmacological protection, or adaptation of an ovarian stimulation protocol for in vitro fertilization (IVF) treatment. The choice of an appropriate method is based mainly on whether a delay in treatment is required, the type of the disease, and the treatment the patient has. Importantly, chemotherapy regimens differ widely in their effects on the gonads. For example, chemotherapy regimens such as vincristine and fluorouracil carry a small risk, whereas other regimens, such as conditioning of the bone marrow for transplantation with busulfan, induced premature ovarian failure in more than 90% of the cases studied, even during childhood [2, 3].

To address the question of fertility preservation management, oncology centers must build a close collaboration with fertility units. In turn, the fertility units themselves must be able to manage these patients with minimal delay and offer the optimal option for each case. Although many centers throughout the world have developed strategies to propose fertility preservation to their patients, increased efforts must be made to inform and to offer adequate advice to the patients regarding their risk of future infertility and the option of fertility preservation. A recent review reported that from 34% to 72% of cancer survivors recalled being counseled by a health provider regarding the impact of cancer treatments on fertility [4]. In addition to the fear of losing their fertility, cancer evokes the possibility of death leading to emotional and psychological distress. While addressing the issue of fertility preservation, patients are encouraged to think about their future, which may help them focus their energies in a positive direction.

This paper aims to discuss the major concerns regarding the risk of premature ovarian failure after cancer therapy and the options for preserving fertility in these patients.

## 2. Effect of Cancer Treatment on Fertility

Human females are born with a fixed and nonrenewable number of primordial oocytes that represent the so-called ovarian reserve. Histological studies and mathematical models have determined that approximately 1,000,000 oocytes are present at birth. At menarche, the number of primordial oocytes has decreased to approximately 180,000 and this number continues to decline over time with fewer than 1000 oocytes remaining at menopause [5]. Oocyte growth, differentiation, and apoptosis are strictly regulated through autocrine and endocrine loops, with molecular mechanisms that have recently been elucidated in the Nos3-knockout mouse model [6, 7]. The pituitary gland produces FSH and LH in response to LHRH released from the hypothalamus, whereas estrogen and progesterone produced by the granulosa and theca cells, respectively, induce a negative feedback on LH and FSH production.

Through the partial or total destruction of the ovarian reserve, cancer treatments may temporarily or definitively affect the ovarian function. The most frequent neoplasms of the reproductive age are breast cancer, malignant lymphomas, malignant melanomas, and gynecological cancers, with an overall incidence of 82.7 cases per 100,000 [8]. Surgery, radiotherapy, and chemotherapy, together with recently isolated target-oriented molecules, have significantly improved the prognosis of young cancer patients. Thus, quality-of-life issues have become a high priority on the patients' agenda. Premature menopause and irreversible sterility are the most dramatic outcomes of ovarian dysfunction; however, infertility and low estrogen levels are associated with an impaired quality of life and severe psychological consequences. Thus, infertility and premature menopause are relevant issues for young women with cancer and may also influence their treatment compliance [9].

The mechanism via which chemotherapy impairs ovarian function has not been completely elucidated. It has been established that drugs have varying effects on ovarian function, with alkylating agents being the most toxic.  Table 1 represents the degree of ovarian toxicity of specific drugs used for the treatment of cancer during reproductive ages. As expected, the total dose is directly correlated to the ovarian dysfunction [10]. Genetic polymorphism within the metabolic pathway of cyclophosphamide activation accounts in part for the different toxicities observed in different individuals [11]. The patients' age is another variable that accounts for the probability of ovarian dysfunction after chemotherapy. Young patients have a higher absolute number of primordial oocytes and have a lower rate of ovarian toxicity after chemotherapy. Nonetheless, young patients face a sharp reduction of their ovarian reserve after toxic chemotherapy, and it has been demonstrated that menopause occurs earlier in cancer survivors who have received chemotherapy in early adulthood [12, 13].  Table 2 summarizes the rate of chemotherapy-induced amenorrhea, segregated by different regimens used in breast cancer, according to the patients' age.

The targets of chemotherapy-induced ovarian toxicity include primordial oocytes, granulosa cells, and ovarian stroma. Familiari et al. demonstrated the direct destruction of primordial oocytes and follicular depletion in ovaries of young patients treated with regimens containing alkylating agents for Hodgkin's lymphoma [14]. Meirow et al. described a similar situation in women who were exposed to nonsterilizing doses of chemotherapy [15]. Oktem and Oktay used an ovarian xenograft model to characterize the impact of chemotherapy on human primordial follicles. They reported apoptosis of primordial oocytes and growing follicles 12 and 24 hours after cyclophosphamide exposure, with a >90% reduction of follicle density 48 hours after treatment [16]. 

Meirow et al. also reported significant vascular damage in histological sections of human ovaries exposed to chemotherapy. They described thickening and hyalinization of cortical vessels, cortical proliferation of small vessels, and focal cortical fibrosis with segmental collagen deposition [15]. These alterations of the normal ovarian anatomy could be a consequence of drug-induced endothelial damage, followed by subsequent neovascularization. Another potential mechanism of primordial oocyte loss is explained by the follicular burnout theory. When exposed to chemotherapy, growing follicles are destroyed, and therefore the amount of inhibitory paracrine factors, in particular anti-Müllerian hormone (AMH), is reduced. These changes induce continuous and sustained recruitment of primordial follicles, thus, “burning out” the ovarian reserve. To date, there is no proof of this theory in humans, although AMH-knockout mice have an increased activation of primordial follicles with a greater number of atretic large follicles and a reduced ovarian reserve [17]. 

Little information regarding the ovarian toxicity of newly available drugs for the treatment of young women with neoplasms is available. For example, trastuzumab is a humanized IgG1 monoclonal antibody that recognizes the extracellular domain of c-erbB2 receptor. Although c-erbB2 is involved in many different cellular processes, no ovarian toxicity has been reported even after prolonged use [18]. Conversely, monoclonal antibodies targeting VEGF are able to impair the normal ovulation process in which angiogenesis plays an important role; however, the data regarding this affect in humans are not available. Imatinib is a small molecule that specifically targets c-Abl and c-Kit in chronic myeloid leukemia (CML) and gastrointestinal stromal tumors (GISTs), respectively. This drug has substantially improved the prognosis of patients affected by CML and GIST and has been proposed as a gonad protector for patients exposed to chemotherapy. In experimental models, DNA damage induces TAp63 phosphorylation via c-Abl, with subsequent cell death. Imatinib inhibits TAp63 phosphorylation in primordial oocytes, thus the oocytes are protected from apoptosis [19]. Inhibitors of m-TOR (mammalian target of rapamycin) have been used for the treatment of kidney cancer and more recently for the treatment of neuroendocrine tumors. In men, m-TOR inhibitors induce severe but reversible azoospermia; however, no data are available for the effects of m-TOR inhibitors on women [20]. 

In parallel with the development of a fertility preservation program, the evaluation of the gonadotoxicity of all new therapeutic regimens should be systematically taken into consideration to adequately inform and manage young patients who are particularly concerned regarding their future fertility. 

## 3. Obstetrical Outcome of Cancer Survivors

In addition to difficulties with conception due to damage of the ovaries, long-term female cancer survivors may also face specific obstetrical risks. Additionally, concerns have emerged regarding possible health problems in the offspring. 

Adverse events such as miscarriages, preterm delivery, neonatal low-birth weight, and postpartum hemorrhage may occur more frequently in cancer survivors than in the general population, depending on the age at treatment and of the type, dosage, and schedule of the anticancer treatment [21–23]. 

While chemotherapy has a major impact on ovarian function, it rarely directly affects pregnancy outcomes [22]. However, the long-term side effects of some chemotherapy regimens may increase the risk of adverse obstetrical and perinatal outcomes. For example, patients treated with anthracycline or >30 Gy of mediastinal radiation may suffer from peripartum cardiomyopathy and should be monitored for cardiac status before pregnancy. However, in a cohort of 53 childhood cancer survivors, none developed anthracycline-induced clinical heart failure, thus the risk of this complication appears to be low [24]. 

Although chemotherapy does not affect uterine function, radiotherapy results in different degrees of uterine damage depending on the total dose and the site of irradiation. When administered in children, pelvic or total body irradiation (TBI) may result in impaired uterine development. The data from the Childhood Cancer Survivor Study, based on more than 2,000 children born from cancer survivors, showed an overall increased risks for preterm births (21.1% versus 12.6% for the children born from a random sample of the cancer survivors' nearest-age full sibling included in the study as controls; OR = 1.9; 95% CI = 1.4 to 2.4; *P* < 0.0001). In this study, the risk differed depending on the type and dose of anticancer treatment (i.e., alkylating, nonalkylating, or radiotherapy treatment) [21]. The obstetrical risk is particularly high for patients subjected to pelvic radiation of >500 cGy; preterm delivery approaches 50% compared with 19.6% for those who did not receive any radiation treatment (OR 3.5, 95% CI = 1.5 to 8, *P* < 0.003). Additionally, these babies had lower birth weights (36.2% versus 7,6%, OR = 6.8, 95% CI = 2.1 to 22.2: *P* < 0.0001) and a higher percentage were small for gestational age (18,2% versus 7,8% OR = 4, 95% CI = 2.1 to 22.2, *P* < 0.001). Furthermore, even patients exposed to lower doses of uterine radiotherapy have a risk for preterm delivery (from 50 cGy) and low birth weight (from 250 cGy). These data were corroborated by Green (2009) on more than 4,000 pregnancies from the same database after inclusion of a larger number of cancer survivors, underlying the lack of evidence of increased risks of simple malformations, cytogenetic syndromes, or single-gene defects in the offspring [22]. 

Additionally, an increased risk of hypertension complicating pregnancy, fetal malposition [25], and post-partum hemorrhage [23] has been reported in patients receiving direct uterine radiotherapy, as observed with Wilm's tumor patients. 

Treatment via estrogen supplements may be helpful, as it has been demonstrated that in women treated with total body irradiation, hormone replacement therapy significantly increases uterine volume and endometrial thickness as well as reestablishing uterine blood flow [26]. Unfortunately, established hormonal replacement therapy is not as effective for prepubertal childhood cancer survivors in rescuing their uterine function. 

Thus, when counseling a patient who desires to conceive after anticancer therapy, evaluation of the type and total dose of treatment received and the age at treatment should be considered to estimate the obstetrical risks, with special concern for patients treated with radiation. 

## 4. Cryopreservation of Ovarian Tissue

Cryopreservation of the ovarian cortex is one of the principal techniques utilized for preserving fertility. Indeed, this established technique has recently been identified as highly promising for this indication. 

The first studies on cryopreservation of the ovarian cortex were performed on rats in the 1950s [27]. In 1960, Parrott was the first to obtain live young from a mouse after ovary removal, cryopreservation, and reimplantation. However, she noted a large decrease in the number of viable oocytes upon necropsy. This work involved the first pregnancies and live births following the freezing of oocytes [28]. Paradoxically, despite this positive result, this work also marked the end of research on this subject for a considerable period. It was not until 1990 that interest in the freezing of ovarian tissue reemerged, with the work of Carroll on the freezing of primary follicles from mice [29]. The protocol used by Carroll, based on a gradual decrease in temperature, was similar to the protocols used for the freezing of embryos, with dimethylsulfoxide used as a cryoprotective agent. In mice, this technique allowed the survival of oocytes (80%) and of the surrounding somatic cells (65%). Offspring were obtained after freezing isolated follicles, thawing, and transplantation to the ovarian bursa of host animals [30]. 

The same freezing protocol has been used for fragments of ovary from ewes, leading to the birth of a lamb after an autologous transplantation [31]. 

The first work on cryopreservation of the human ovarian cortex was published in 1996. The various freezing protocols tested were compared with that described by Carroll et al. [29]. Hovatta provided the first demonstration that the freezing of human ovarian cortex was feasible [32]. After thawing and histological analysis, freezing and thawing were found to have had no effect on the number of follicles, follicle diameter, or oocyte diameter, regardless of the cryoprotectant agent used (DMSO- or PROH-sucrose). In the same year, Newton et al. compared the effects of four cryoprotectant agents (DMSO, ethylene glycol (EG), PROH, and glycerol) on the survival of human ovarian tissue after freezing. Follicular survival rates were the highest with EG (84%) and the lowest with glycerol (10%), with those for DMSO (74%) and for PROH (44%) falling between these values [33]. These studies also showed that freezing protocols similar to those used to obtain a live lamb from an ewe—a large mammal in which ovary organization resembles that in women—through freezing/thawing and the autologous transplantation of ovarian tissues were transposable to humans. 

Thus, in the mid-1990s the idea first emerged from preserving the fertility of patients or from creating oocyte banks through freezing ovarian tissue to treat infertility [34]. It became clear that cryopreservation of the ovarian cortex was potentially useful for patients having to undergo highly gonadotoxic treatments because it would be possible to preserve a large number of primordial follicles. Of note, the first reported case of ovarian cortex cryopreservation in a patient was published in 1996 [35]. 

Cryopreservation of the ovarian cortex has since become an integral part of fertility preservation, and results from several cohort studies have been published [36–39].

**Indications**

Ovarian cryopreservation involves the removal of all or part of one of the two ovaries and thus, the excision of a substantial proportion of the follicular capital of the patient. Therefore, cryopreservation should only be offered to patients undergoing highly gonadotoxic treatments. The various treatments have been classified according to their toxicity to the ovaries [40, 41]. The age of the patient must also be taken into account, as the impact on the ovarian reserve is lower in younger patients. It would therefore be justified to offer ovarian tissue cryopreservation in three situations in which it is almost certain that ovarian function will be altered at the end of treatment: chemotherapy with high doses of alkylating agents (such as busulfan or cyclophosphamide); total body irradiation or abdominal radiation; bilateral ovariectomy or unilateral ovariectomy of a single-remaining ovary. Ovary cryopreservation may also be offered in cases of disease associated with premature ovary failure, such as Turner syndrome [42, 43].

This technique has many advantages. In addition to allowing the preservation of a large number of immature oocytes within primordial follicles, this technique does not require ovarian stimulation and can be organized very rapidly without the need to defer treatment of the disease. It is available to patients regardless of their marital status and is the only possible technique for fertility preservation in prepubescent girls [2, 36]. By contrast, an upper age limit of 35 to 37 years is recommended for the cryopreservation of ovarian tissue [44]. The risks of ovary tissue harvesting appear to be small: only a single patient was reported to have required surgical revision among the 500 cases of ovary cryopreservation described by the FertiPROTEKT network [44].

In practice, ovarian tissue is usually obtained via laparoscopy. However, it may be collected via laparotomy, particularly from patients treated for neuroblastoma. In these cases, the ovary can be harvested during resection of the residual tumor, a procedure scheduled prior to high-dose chemotherapy and the autologous transplantation of hematopoietic stem cells. The quantity of tissue removed is variable; part or all of an ovary may be removed or ovarian tissue may be excised via biopsy [45].

The operating theater is rarely adjacent to the laboratory in which the ovarian tissue is frozen and stored. Therefore, the ovary must be transported in optimal conditions to the site of freezing immediately after its removal. Most authors recommend that transport times should be kept as short as possible. However, a series of five pregnancies following the autologous transplantation of frozen and thawed ovarian tissue has been reported, including two cases in which the fragments of ovarian tissue were frozen only after transport periods of four to five hours [46].

The penetration of cryoprotectant agents into the tissues is limited. Isolation of the ovarian cortex through the removal of the medulla is therefore important, to reduce the thickness of the ovarian tissue, thereby optimizing follicular survival [47]. After a period of equilibration with a cryoprotective agent, the ovarian fragments are frozen in a slow-freezing protocol. Indeed, all the pregnancies achieved to date have involved the slow freezing of ovarian tissue.

Two major developments are currently the subjects of a heated debate: the vitrification of ovarian tissue fragments [48, 49] and the freezing of the entire ovary [50, 51]. It has recently been shown that the ultrastructure of human follicles is conserved during vitrification in a closed system based on cryotubes [52]. Freezing of the whole ovary has been proposed as a solution for limiting the phenomenon of follicular loss due to ischemia during ovarian cortex fragment revascularization following autologous transplantation. Most relevant studies have been performed in the ewe, despite the ovary being smaller in lambs and differences in the anatomy of the vascular pedicle. The technique involves the perfusion of ovarian vessels with freezing solution, then placing the ovary in a tube containing a similar solution. The efficacy of this technique was demonstrated for the first time in 2004. The transplantation of an entire ovary that had been frozen in DMSO allowed the revascularization of the ovary using microsurgical reanastomosis in five of eight ewes (62.5%). Normal estrus cycles were reestablished in three of the ewes, as demonstrated via progesterone levels [51]. Oocytes have been obtained from frozen whole ovaries in ewes [53], although this procedure is inefficient (after autologous transplantation only, 25% of the ovaries were functional), and a single birth has been achieved using this method [54].

Only few studies have reported the freezing of an entire human ovary. No significant difference in follicular viability between whole frozen ovaries and frozen ovary fragments was observed [50]. These results have since been confirmed by Martinez-Madrid et al. [55].

Finally, ovary harvesting could also make it possible to develop additional techniques for preserving fertility. Indeed, the freezing of ovarian tissue may be combined with the removal, via puncture, of small antrum follicles that may be present in the ovary at the time of harvesting. This makes it possible (1) to freeze ovary tissue and isolated immature oocytes [42]; (2) to freeze isolated oocytes present in the dissection medium when ovary fragments are generated [56]; or (3) to freeze pre-antral follicles present in the medulla, where they may be highly abundant [57].

## 5. Transplantation of Ovarian Tissue

At present, ovarian tissue grafting is the only option for potentially restoring ovarian function and fertility after cryopreservation and storage of ovarian tissue. Different groups throughout the world have published the results of successful transplantation procedures leading to the reactivation of the ovarian endocrine activity and ovulation [58]. 

Despite these promising results, the technique should not be considered a routine clinical procedure, as there have been fewer than 20 babies born through this method in the last decade [59]. It is difficult to perform statistical analysis on the efficiency of the procedure because a limited number of patients underwent ovarian autotransplantation compared with the number of patients who cryopreserved their ovarian tissue. A pregnancy rate per transplantation ranging from 20% to 30% appears to be realistic, although the delivery rate may be lower [59–61]. 

Different protocols have been applied to perform the grafting, with a wide variability concerning the site of the transplantation [62]. The ovarian tissue is able to restart folliculogenesis up to ovulation when grafted either to an orthotopic site (ovarian fossa, remaining ovary) or to a heterotopic site (e.g., peritoneal abdominal wall, uterine serosa, subcutaneously on the abdominal wall or in the forearm). Despite the fact that ovulation, egg retrieval, in vitro fertilization, and early-stage embryo development can occur after heterotopic transplantation and lead to biochemical pregnancy, ongoing pregnancy and delivery have been obtained only after orthotopic graft at the ovarian site [63–66]. After the transplant, variability has been observed in the time needed to restore ovarian activity and in the lifespan of the graft. Follicular development generally occurs in 4 to 5 months because at least 120 days are necessary to initiate follicular growth, but it has been observed to occur in a wide window between 8 and 26 weeks [60]. The graft can be functional from 3 to 4 months up to more than five years (personal data). Several factors may explain these important clinical variations, such as the original follicle density in the ovarian tissue, the amount of tissue transplanted, the age of the patient at the time of cryopreservation, the freezing/thawing technique, the ischemic injury at the time of the graft before neoangiogenesis occurs, and the hormonal environment [62]. 

Based on these observations, various strategies have been suggested to optimize the function of the transplant, such as graft of isolated follicles, extremely thin ovarian fragments [67, 68], or pretreatment of the host and graft with hormones, vitamins, and growth factors [69, 70]. Moreover, orthotopic and heterotopic transplant of the whole ovary with vascular anastomosis of the pedicle vessels was performed successfully in humans with fresh organs in cases of pelvic irradiation or allotransplantation of monozygotic twins discordant for ovarian failure [71–74], but further investigations on cryopreservation of the entire ovary are needed in both humans and animal models. 

A major concern when transplanting ovarian tissue to a patient treated for oncological disease is the possible risk of reintroduction of cancer cells within the graft. This risk is different depending on the type and stage of the original cancer. Different authors have classified pathology as low, intermediate, and high risks of recurrence in cases of transplant to drive their decision; for example considering Hodgkin's lymphoma as low risk, breast cancer as intermediate risk, and leukemia as high risk [75]. 

As transplantation is the only option available at present to restore fertility using cryopreserved ovarian tissue, each patient should be clearly informed regarding the safety issue and the limitations of ovarian tissue transplantation procedures at the time of the fertility preservation counseling. 

## 6. In Vitro Maturation and Oocyte Vitrification

Vitrification of oocyte and embryos is a well-established procedure and is currently performed in many IVF laboratories with a high success rate. This procedure should be considered as a first option for fertility preservation, before gonadotoxic treatment, when feasible. However, stimulation protocols for oocyte or embryo freezing in cancer patients present two main safety issues. 

The first concern is the exposure to superphysiological estrogen levels, which may be 10–20 times more than those observed in natural cycles [76, 77]. Although the effect of a temporary increase in estradiol levels on the risk of the recurrence of cancer is uncertain, it remains a concern in cases of hormone-dependent tumors [78, 79]. 

The second concern is that the duration of the treatment may delay the start of the chemotherapy. Due to the risks inherent in the malignant disease, fertility preservation cannot postpone the treatment course. 

Recently, emergency procedure of random start-controlled ovarian stimulation in the late follicular phase or in the luteal phase have been reported with success, significantly shortening the time required to undergone oocyte or embryo cryopreservation before chemotherapy [80]. Furthermore, this treatment can be associated with letrozole administration to reduce the effect of temporary increase of the oestradiol level in the case of hormone sensitive tumor [81]. 

Another strategy to avoid those safety issues is the use of in vitro maturation (IVM) of oocytes and vitrification. 

### 6.1. Cryopreservation of Oocytes

During cryopreservation procedure, three major mechanisms may potentially damage the cell, including chilling injury (+15 to −5°C), ice crystal formation (−5 to −80°C), and fracture damage occurring from solidified fluid within the cell (−50 to −150°C). During warming stages, the cell is subjected to the same injuries in reverse order [82]. Two techniques are used in assisted reproduction laboratories for cryofreezing; controlled slow freezing (SF) and, more recently, an ultrarapid cooling method also known as vitrification. Both involve the use of cryoprotectants to minimize ice-crystal formation [82]. 

Specific characteristics unique to oocytes make them more susceptible to injuries induced by slow freezing; these characteristics include their size, shape, and water content. These injuries result in spindle and cytoskeleton damage, crystal formation, and zonal hardening. Those changes explain the lower fertilization and implantation rates observed with frozen-thawed oocytes [83]. 

Vitrification was introduced as a means to protect against the ice-crystal formation that has been observed with slow cooling. The tissue is submerged directly into liquid nitrogen after being treated with high concentration of cryoprotectant, and freezing occurs without allowing crystal formation [84]. Recently, the use of low concentrations of the cryoprotectants offsets their observed toxicity towards oocytes [85]. Furthermore, ICSI and improvements in culture media have helped overcome other side effects of vitrification, such as zonal cracks and hardening. Thus, a steady increase in oocyte survival, fertilization, and pregnancy rates has been observed. Pregnancy rates after oocyte vitrification have been reported in the 10–30% range [86, 87]. In a meta-analysis by Cobo and Diaz in 2011, vitrification methods demonstrated a higher fertilization rate, cleavage rate, and optimal quality rate of the embryo compared with slow freezing [88]. At the MUHC Reproductive Center, we reported a mean oocyte survival rate of 81% after thawing, a 76% fertilization rate per oocyte, a clinical-pregnancy rate per cycle of 45%, a live-birth rate of 40%, and the birth of 22 healthy babies [89]. By 2009, nearly 1,000 live births were reported from oocyte cryopreservation with an incidence of congenital abnormalities in these children of 1.3%, similar to that observed in the general ART population [90]. 

### 6.2. In Vitro Maturation of Oocytes

Due to a concern regarding hormonal sensitivity, gonadotropin treatment could be avoided via the collection of immature oocytes in unstimulated cycles for fertility preservation [91, 92]. This procedure results in the successful preservation of fertility with no delay in the administration of chemotherapy, no surgery, and a lack of the necessity of gonadotropin stimulation. 

The oocyte collection is performed 38 hours after hCG priming, ideally when the largest follicle has reached 10–12 mm [93, 94]. If there is no sufficient time prior to chemotherapy for conventional follicular phase oocyte retrieval in a stimulated or unstimulated cycle, retrieval in the luteal phase could be considered [95, 96]. After collection, the oocyte maturation status is assessed; immature oocytes are cultured in IVM medium and periodically assessed for maturation status as previously described [93]. When the female patient does not have a stable partner and does not wish to use donor sperm to create embryos, she can opt for oocyte cryopreservation. However, vitrification of oocytes matured in vitro remains in the experimental phase, as only a 20% live-birth rate per cycle from vitrified of IVM oocytes has been reported [89] due to the significantly lower survival and fertilization rates compared with oocytes collected following ovarian stimulation [94]. Notably, these studies demonstrated that vitrification of in vitro matured oocytes collected from unstimulated cycles followed by later thawing and fertilization can result in successful pregnancies and live births. In 2009, we published a report of the first 4 live births achieved after vitrification and thawing of in vitro matured oocytes [86]. 

Although it was expected that smaller immature oocytes would have an increased survival rate when undergoing the cryopreservation procedure, the potential of oocyte maturation is reduced by the vitrification at the GV stage [97]. Therefore, immature oocytes are currently matured in vitro prior to vitrification. 

After thawing, ICSI was routinely performed 2–4 hours after polar body extrusion due to a theoretical risk of zonal hardening during the in vitro culture period [98]. 

The main disadvantage of IVM concerns lower pregnancy rates when compared with conventional IVF due to lower implantation rates [99]. This has been overcome through transferring one embryo more than in IVF, which allows comparable pregnancy rates to those of IVF to be attained (McGill, unpublished data). 

Safety issues of IVM have been evaluated in several studies. Buckett et al. compared congenital anomalies in 432 patients undergoing IVF (217), IVM (55), and ICSI (160) with non-ART-conceived children [100]. There was no increase in congenital anomalies after IVM when compared with spontaneously conceived children. A recent retrospective study also observed no increase in chromosomal abnormalities in 6 children born after IVM when compared with 30 children born after classical IVF [101]. Those initial reports are encouraging, though few in numbers. Thus, the safety of IVM must be confirmed with larger-scale studies. 

To date, the MUHC Reproductive Center has provided fertility preservation to 183 patients with breast, hematological, brain, soft-tissue, colorectal, and gynecological cancers. Of these, 128 patients underwent oocyte retrieval without ovarian stimulation followed by IVM. 

## 7. Pharmacological Protection

Pharmacological protection of the gonads during chemotherapy constitutes an attractive option to preserve fertility. It may allow the restoration of normal ovarian function and natural fertility after treatment and may therefore save the patients from adverse events related to premature ovarian failure. 

The gonadotropin-releasing hormone agonist (GnRHa) is a decapeptide derived from the native hormone; however, this synthetic hormone binds to specific pituitary receptors with a higher affinity. The sustained-release of GnRHa initially stimulates the release of gonadotropins, inducing a brief ovarian hyperstimulation, commonly known as the flare-up response. After 10–15 days, pituitary GnRH receptors are downregulated and the inhibitory effect of the synthetic hormone on ovarian function is observed. During treatment, the hormonal profile is characterized by low gonadotropin and estradiol levels. This inhibitory effect may protect the ovarian function during chemotherapy through different mechanisms, such as maintaining the follicles at the primordial/primary stages, decreasing sensitivity to gonadotoxic treatment, or through the reduction of the blood supply. 

The effectiveness of the treatment was first reported in rats and monkeys in the 1980s. Administration of GnRha during chemotherapy has proven to reduce follicular depletion in rats treated with cyclophosphamide [102, 103]. Experiments using monkeys demonstrated that 65% of the primordial follicular pool is destroyed after cyclophosphamide treatment compared with 29% with Gn-Rha cotreatment [104]. Although this protective effect in animals has been supported by another study [105], this effect has been reported as limited or dose dependent [106] and not sufficient to protect fecundity by others [107]. The efficiency of GnRh antagonist in inducing an immediate ovarian suppression through competitively blocking GnRh receptor in the pituitary has also been investigated with divergent conclusions according to the published reports [108–110]. 

In humans, the efficiency of GnRha in preventing premature ovarian failure was controversial for years and is still debated. Nonrandomized studies suggested a reduction of premature ovarian failure rate when GnRha was administered concomitantly to the chemotherapy [111, 112]. However, the methodology of these studies was criticized, thus calling the results into question [113, 114]. Waxman et al. first reported the results of a prospective, randomized study on a cohort of 18 patients and failed to demonstrate any significant protective effect of GnRHa treatment after two years of followup [115]. In a cohort of 80 randomized patients treated for breast cancer, Badawy et al. reported a reduction in premature ovarian failure rate with GnRha [116]. However, mean FSH values were lower than 20 IU/L in both groups and FSH value defining premature ovarian failure was not indicated. Furthermore, the data were collected after less than 1 year of followup. 

Recently, GnRha cotreatment failed to prevent premature ovarian failure in a small cohort of patients with Hodgkin's lymphoma treated with a regimen of escalated BEACOPP (bleomycin, etoposide, adriamycin, cyclophosphamide, vincristine, procarbazine, and prednisolone) [117]. The ZORO study reported no significant differences in the restoration of the spontaneous cycle and hormonal profile after GnRha co-treatment compared with control in patients up to 45 years of age treated for breast cancer [118]. However, possible biases were highlighted regarding the results of this study [119]. A recent meta-analysis suggests a potential benefit of GnRha treatment during chemotherapy on spontaneous resumption of menses and ovulation, but not on pregnancy rate. The authors of this meta-analysis also moderated their conclusion by listing the risk of bias in the published trials assessing this issue [120]. The largest randomized study of 281 early breast cancer patients was recently published by del Mastro et al. (2011), showing a reduction in the occurrence of early menopause in the group of patients with breast cancer treated with GnRha during chemotherapy after 1 year of follow-up [121]. In contrast, the efficiency of triptorelin to prevent premature ovarian failure after 1 year of follow-up was not observed in a cohort of 80 randomized young patients treated for lymphoma [122]. 

Thus, despite the potential efficiency of GnRha to prevent premature ovarian failure suggested by some of the studies, the results must be confirmed in the future. Additionally, the effect of GnRha on the fertility of the patients must be further investigated. GnRha treatment may also have additional advantages that should be taken into consideration, such as reducing the risk of thrombocytopenia-associated menorrhagia during chemotherapy [123]. GnRha treatment during chemotherapy is actually proposed as an option to preserve fertility in many centers worldwide. However, this option should be offered as part of a clinical trial after careful counseling regarding the other existing available options to preserve fertility. 

Globally, a new concept of “fertoprotective adjuvant therapy” has arisen. This concept supports that molecules could be developed to directly prevent DNA damage caused by chemotherapeutic agents on gonads without interfering with their efficiency. A more thorough understanding of the mechanism of oocyte damage and DNA repair during chemotherapy is essential to address this question. In mice, oocytes lacking the gene for acid sphingomyelinase or wild-type oocytes treated with sphingosine-1 phosphate-resisted apoptosis induced through anticancer therapy [124]. Sphingosine 1-phosphate also led to the preservation of fertility in irradiated female mice without propagating genomic damage to the offspring [125]. Other proteins involved in apoptosis, such as Bax and Rad51, play a critical role in oocyte death after doxorubicin treatment or during age-related physiological oocyte depletion [126]. Experiments in mice show that AS101, a nontoxic immunomodulator, can specifically protect against cyclophosphamide-induced damage in the testis [127]. 

Recently, a new potential protective drug, imatinib (trade name Gleevec), was also investigated in cisplatin-treated mice [19]. These results offer a promising approach for the use of chemotherapy without harming the resting germ cell [128]. 

This important ongoing experimental research may contribute to the development of new protective candidate therapies targeting individualized chemotherapeutic agents. 

## 8. Conclusion

The option to preserve fertility must be chosen after multidisciplinary counseling for each patient. Each option presents advantages or contraindications that must be taken into consideration; however, none of them guarantees the success of the procedure. Thus, the combination of different techniques may offer the best chance to restore the fertility of the patients in the future. 

The combination of ovarian tissue cryobanking and immature oocyte collection from the tissue followed by IVM and vitrification of the matured oocytes (or embryos) represents a promising approach for fertility preservation. Furthermore, these immature oocytes can be collected at any time of the cycle. Recently, preantral follicle isolation from the medulla during ovarian tissue cryopreservation was also reported, offering additional materials for storage. When feasible, a classical IVF protocol with prior gonadotropin stimulation should be proposed, as it remains the only established procedure for storing gametes. 

Finally, reducing the gonadotoxicity of the treatment by adapting the chemotherapy regimen, protecting ovaries from irradiation, or using pharmacological protective therapy can also be proposed associated with a cryopreservation procedure. 

## 9. Disclosure

The co-authors are listed alphabetically and equally contributed to the paper.

## Figures and Tables

**Table 1:** Risk of ovarian toxicity of antineoplastic drugs.

High risk	Cyclophosphamide, ifosfamide, busulfan, mechlorethamine, melphalan
Intermediate risk	Doxorubicin, epirubicin, cisplatin, docetaxel, paclitaxel

Low risk	Vincristine, methotrexate, 5-fluorouracil, trastuzumab

**Table 2:** Rate of chemotherapy-induced amenorrhea, according to regimen and age.

Regimen	Age <30 yrs	Age 30–40 yrs	Age >40 yrs
No treatment	1%	< 5%	20–25%
Ac x4	—	13%	60%
CMF x6	19%	35%	85%
CAF/CEF x6	30%	—	85%
AC x4->Dtax	—	55%	—

AC: doxorubicin/cyclophosphamide,

CMF: cyclophosphamide/methotrexate/5-fluorouracil,

CAF: cyclophosphamide/doxorubicin/5-fluorouracil,

CEF: cyclophosphamide/epirubicin/5-fluorouracil,

Dtax: docetaxel.
